# Lean Management—The Journey from Toyota to Healthcare

**DOI:** 10.5041/RMMJ.10107

**Published:** 2013-04-30

**Authors:** Sorin T. Teich, Fady F. Faddoul

**Affiliations:** Associate Professor, Assistant Dean of Clinical Operations, Department of Comprehensive Care, Case Western Reserve School of Dental Medicine, Cleveland, Ohio, USA; and; 2 Professor, Director of AEGD, Department of Comprehensive Care, Case Western Reserve School of Dental Medicine, Cleveland, Ohio, USA

**Keywords:** Lean management, Pareto, waste, continuous improvement, healthcare, quality, customer value

## Abstract

The evolution of production systems is tightly linked to the story of Toyota Motor Company (TMC) that has its roots around 1918. The term “lean” was coined in 1990 following the exploration of the Toyota model that led to the “transference” thesis sustaining the concept that manufacturing problems and technologies are universal problems faced by management and that these concepts can be emulated in non-Japanese enterprises.

Lean is a multi-faceted concept and requires organizations to exert effort along several dimensions simultaneously; some consider a successful implementation either achieving major strategic components of lean, implementing practices to support operational aspects, or providing evidence that the improvements are sustainable in the long term.

The article explores challenges and opportunities faced by organizations that intend incorporating lean management principles and presents the specific context of the healthcare industry. Finally, the concepts of “essential few” and customer value are illustrated through a simple example of process change following lean principles, which was implemented in a dental school in the United States.

## HISTORY OF THE LEAN CONCEPT

The evolution of production systems is tightly linked to the story of Toyota Motor Company (TMC) that has its roots around 1918 when Sakichi Toyoda, who held a patent for an automatic loom that revolutionized the weaving industry, established his business. After selling the patents in 1929, the company reinvented itself in the automotive industry that, at the time, was dominated in Japan by local subsidiaries of Ford and General Motors (GM). Truck and car production began in 1935, and in 1937 TMC was formally incorporated.

By 1950, the entire Japanese auto industry was producing an annual output equivalent to three days of the US car production; it was around this time when Eiji Toyoda was sent to the US to study manufacturing methods. Another valued TMC employee, Taiichi Ohno, who joined the company in 1943, joined the visit and reasoned that the Western production systems had two major flaws^1^:

1. Producing components in large batches resulted in large inventories, and
2. The methods preferred large production over customer preferences

Little by little, through much iteration, the Toyota Production System (TPS) evolved and provided a tool that used innovation and common knowledge, and that functioned well in an environment with different cultural values compared with the Western hemisphere. Only in 1965, when the system was rolled also to TMC’s suppliers, TPS began to be documented, and it was largely unnoticed until 1973 when the oil crisis affected the global automotive industry.

The performance gaps between Toyota and other car-makers were highlighted in 1990 in the book *The machine that changed the world*,^2^ in which the term “lean” production was coined. The exploration of the Toyota model led the authors to postulate the “transference” thesis that sustained the concept that manufacturing problems and technologies are universal problems faced by management, and that these concepts can be emulated in non-Japanese enterprises. In the next few years, the process of “extension” was accelerated by reports of Western companies in diverse sectors, incorporating lean principles that involved^3^–^5^:

1. Identification of customer value
2. Management of “value stream”
3. Developing capabilities of flow production
4. Use of “pull” mechanisms to support flow of materials at constrained operations
5. Pursuit of perfection through reducing to zero all forms of “waste”

Customer value identification was crucial in moving away from a production floor focus towards an approach that sought to enhance this value by adding product/service features while eliminating wasteful activities. As such, value is related to customer requirements, and it will be the customer that ultimately determines what constitutes *muda* (waste in Japanese) and what does not.

Lean is a multi-faceted concept and requires organizations to exert effort along several dimensions simultaneously; some consider a successful implementation achieving major strategic components of lean, implementing practices to support operational aspects, and providing evidence that the improvements are sustainable in the long term.^6^

Clearly, this ambitious approach requires deep commitment and is setting a bar that impacts the organization at all levels. The question is how one can assess if a company is ready for such a drastic change and what it would take in order to ensure a successful transformative process; it is probably easier to provide an answer to the following complementary question: What are the main reasons for failures in companies that tried to implement a lean culture? These were identified as lack of senior commitment, lack of team autonomy, lack of organizational communications, organizational inertia, and lack of interest in lean.^6^–^9^ Another major factor is that lean provides principles for theoretical efficiency that implies more production with a smaller work-force; therefore workers may fear for their jobs.^10^

Recipes for implementation and lessons learned from failures have been reported^6^,^7^; the common threads of these were that organizations need to change at a behavioral and cultural level and this should be translated directly into an endless process of continuous improvement. Despite these being framed in the realm of tangible strategic business direction, “cultural changes” and “endless improvement” are abstract concepts; furthermore, these principles imply that there is no horizon for successfully completing the task because the improvement process is infinite.

Another crucial aspect that should be considered is that lean practices should be considered under the umbrella of their cultural origin. The main three characteristics of Japanese management thinking are harmony and group loyalty, consensus in decision-making, and lifetime employment, all encompassed in the concept of “respect for people.” This concept was not historically understood in the USA where companies only focused on “continuous improvement.”^11^

We submit therefore that the main sources of failures mentioned above are not the technicalities related to lean implementation, but principles that constitute a larger puzzle. Clearly there are stages and steps in implementation of the lean culture, such as prioritizing projects and areas that should be restructured, but the larger picture that implies cultural changes sustaining an endless process may be too intricate for many companies.

So, is lean doomed to be successful only in a handful of companies that are already positioned for the deep structural changes required by this philosophy, or is there a solution that can lead others to benefit from it? Is lean a medicament for the healthcare industry that faces unprecedented technological and financial challenges? In order to address these questions, we have to explore territories that at first glance may seem unrelated.

## THE PARETO PRINCIPLE

The Pareto principle is referred to as the 80–20 rule or the law of the vital few.^12^ The Italian economist Vilfredo Pareto noted around 1906 that 80% of the land in Italy was held by 20% of the population. He confirmed his findings when he analyzed properties in other countries, but, most interestingly, he also noted that the rule also applies in biology; it was Pareto who noticed that 20% of the pea pods in his garden produced 80% of the peas. In time, it became evident that the axiomatic principle applies in economics, customer relations, software development, etc.

In 1937, Joseph Juran stated that this principle also applied to defects, concluding that 80% of the problems are caused by 20% of the defects—and he named this effect the Pareto principle.^9^ A later example was provided by Microsoft that observed that by fixing 20% of the most reported bugs, 80% of the software crushes will be eliminated.^13^

Because most decisions are made under uncertainty,^14^ the vital few must be identified if a program of improvement is to succeed. The importance of the vital few lies in the fact that nothing of significance can happen unless it happens to this (20%) segment.^15^

## LEAN IN THE HEALTHCARE INDUSTRY

It was the same Joseph Juran who linked manufacturing and the healthcare industry; he wrote: “as the health industry undertakes…change, it is well advised to take into account the experience of other industries in order to understand what worked and what has not. … [I]n the minds of many, the health industry is different. This is certainly true as to its history, technology and culture. However, the decisive factors in what works and what does not are the managerial processes, which are alike for all industries.”^16^ This is the reasoning that allows the principles of lean production and management to be applied in healthcare, despite these being originally developed for application in other industries.

We mentioned that the lean philosophy calls for value creation through elimination of waste. These wastes are common in all industries and are not unique to healthcare. The following is a summary of these wasteful activities^16^,^17^:

1. Overproduction—producing something in excess, earlier, or faster than the next process needs it
2. Inventory—the cost of managinga large supply inventory may not be obvious at first glance; beside consumption follow-up and space required to store, there is a need to follow expiration dates and to constantly ensure that the items in the inventory are not technologically obsolete. It was already shown that the overall cost of smaller and more frequent shipments is lower than a large-volume purchase for which a discount was provided
3. Motion—a lot of walking waste can arise from poor design of the working area
4. Transportation—in healthcare this can be evident when moving patients, lab tests, information, etc.
5. Over-processing—there are times when material provided to the customers (patients) mandated by regulations can be confusing. For example, multiple insurance claim forms, including ones that are not bills, can confuse the unexperienced “novice”
6. Defects—there are many examples for these defects that can be related to poor labeling of tests, incomplete information in patients’ charts or in instructions provided to referrals, etc.
7. Waiting—there is not much need to explain why waiting a few hours in line is a wasteful activity
8. Under-utilizing staff—under-use is not only time-dependent but also involves deeper levels such as not sharing knowledge or not taking advantage of someone’s skills and creativity; under-use typically shows in hierarchical structures and not using teams

It was suggested that in order to implement lean in healthcare, the patient has to be the center of the initiative, while time and comfort should be added as key performance measures in the system. Defining the patient as the primary customer requires a conceptual leap because usually the customer pays directly to the enterprise, whereas in healthcare third-party payments depending on the level of insurance are common.^5^ However, if it is understood that value is related to customer requirements and it will be the customer that ultimately determines what constitutes waste, it becomes evident that patients’ demands may require changes even in processes that may not be directly related to patient care.

Early approaches for implementation of lean principles in healthcare were but an exercise to transfer manufacturing principles in order to reduce physical inventories in hospitals,^18^ but later the following types of implementation were reported^19^:

1. Manufacturing-like studies
2. Managerial and support case studies
3. Patient-flow case studies
4. Organizational case studies

Most of these applications (57%) occurred in the USA. The levels of implementation can be defined at three levels^19^:

1. Micro—operational level outcomes represented by manufacturing-like, managerial and support, and patient-flow cases
2. Meso—strategic level that focuses on financial health of organizations, with potential outcomes being financial, staff morale, and involvement
3. Macro—outcomes of national initiatives such as the National Health Service plan in the UK^20^

It was noticed that as implementation of lean principles in healthcare becomes more popular in the USA and Europe, a shift from manufacturing-like to organizational cases is observed in the literature. 11 However, the same study^11^ reports that no publications were found on lean deployment in Japanese healthcare organizations; the authors speculate that this may be a result of either the lack of Japanese case publishing tradition, or the fact that lean is naturally embedded in the Japanese culture and only outstanding cases were reported.

Several examples of successful implementation of comprehensive lean projects in healthcare institutions were reported.^21^–^23^ For example, at Virginia Mason Medical Center (VMMC), where “patient is God,”^23^ the hospital reported increased profit margins, decrease in deaths, and decrease in the number of medication errors. Other reported benefits are an 85% reduction in how long patients wait for a lab result, increased productivity by 93%, and lowering inventory costs by $1 million. In order to reach these results, in 2002, 30 senior managers traveled for two weeks to observe the Toyota Production System at TMC. As the CEO mentioned, among the lessons learned was that “the institution didn’t fall apart without us.”^23^ Since then and until 2008, more than 200 employees have toured production plants in Japan.^21^

Challenges towards lean implementation in healthcare are related to the concepts of value, metrics, and evidence.^24^ Evidence shows that healthcare in the USA lacks efficiency, is not patient-centered, does not provide timely services, and is not equitable (the last two being related to many patients being under-insured).^17^ Redesigning such a system around values such as patients being “primary customers,” emphasizing clinical and services outcomes, using evidence-based tools, and adopting rigorous quality improvement methods may be a phenomenal challenge if it is imposed at the macro or even the meso strategic levels.

We would like to suggest a different approach that promulgates that lean implementation should begin at the microlevel; if a lean project is to be implemented only for a specific area, then the definition of “senior management” will turn out to be the “senior management of the specific area where the implementation is conducted.” A holistic approach that should be connected to the larger context^25^ will rather be a process/unit/department that takes improvement steps, in the context of a larger cluster of departments or the institution. Communication and problem solving will be simple and fast at this level, where mid-level managers have to supervise their dedicated areas, compared with institutional implementation where the CEOs or Chief Medical Officers have to address a myriad of problems overarching the institution. Finally, the customers (patients) of a specific division may have unique characteristics that may not be shared by other patients seen in the institution.

As previously mentioned, resistance to changes is driven in most companies by lack of executive support.^8^ However, following a bottom-to-top approach will redefine the responsibility of the institution’s senior management to three main strategic areas; rather than committing a large amount of time and energy to the lean process, senior managers should:

1. Identify the “vital few” areas that will benefit most from implementing lean. Therefore, the Pareto principle will be applied identifying the 20% areas that will provide 80% of institutional benefits allowing the organization to maximize the return on investment (ROI).
2. Be committed to allocate the supporting resources necessary for the required changes, and, in turn, the area managers will be solely in charge of the process and periodically communicating progress.
3. Be in charge to ensure effectively communicating the results of the changes to the stakeholders in the organization, especially those who are not participating in, or affected by, the lean process. This approach will be also instrumental in institutional cultural change, allowing managers in areas that did not implement lean to observe the benefits incurred at all levels.

Rather than concentrating only on operational aspects of lean thinking, managers at the departmental/unit level will be able to reconcile operations with socio-technical aspects that respect the “human system,” i.e. take into consideration the effects of the changes on the employees with whom they have daily interactions. Therefore, in order to create “cumulative capabilities” and value, managers at all levels need to realize that their job is not only improving the processes, but developing the departmental job-force that ultimately is in charge of the implementation.^3^

## EXAMPLE OF LEAN IMPLEMENTATION AT AN ACADEMIC HEALTHCARE INSTITUTION

Case Western Reserve University School of Dental Medicine (SODM) is located in Cleveland, Ohio and enrolls yearly around 70 students who participate in a 4-year rigorous academic program towards the dental doctoral DMD degree. Clinical training includes third-and fourth-year students providing dental care; the DMD clinic has 143 operatories in which students treat yearly over 8,000 patients throughout over 30,000 encounters. In addition the school has 75 simulation operatories in which first-and second-year students learn clinical procedures on mannequins.

Being an institution that integrates academic education with patient care, one has to define the following customer groups:

1. Students, “primary customers”—who pay tuition and therefore are a source of revenue
2. Patients, “primary customers”—who pay a (reduced) fee for dental treatments
3. Faculty and staff—“secondary customers,” considered by the school as “internal customers” and will be influenced by any changes in processes and policies

Such institutions are in a unique situation because they have more than one group constituting the “primary customers.” In the United States, dental schools are also guided by standards published by the Commission on Dental Accreditation (CODA); these standards do not allude directly to lean strategies but contain statements regarding the obligation to implement “continuous quality improvement” at all levels, quality assurance systems that include cycles of “Plan, Do, Check, Act,” and evaluation and application of new technologies.

It is beyond the scope of this article to present all the tools that support the lean concept; these are presented in great detail in several publications.^16^,^26^,^27^ We present an example of implementation of a new technology in the DMD clinic that illustrates use of some lean instruments.

## ELECTRICAL HANDPIECES

Since its introduction in the nineteenth century, the handpiece has been an integral part of the dental armamentarium. Today, both air-driven and electrical handpieces are available in the marketplace; electrical handpieces^28^ are equipped with a control system that maintains speed as the load on the bur increases.^29^ Electrical technology has several significant advantages over the air-driven handpieces, such as higher torque with little stalling, reduced noise levels, reduced levels of vibration, increased cutting precision and efficiency, and flexibility of use of a variety of handpieces employing the same motor and control box.^30^–^33^ The inherent design of electric handpieces has the potential to reduce contamination by generating less aerosol and allowing less bacterial colonization.^31^

Recent surveys show that there is an increase in adoption of electric handpieces, and around 45% of dentists plan to buy one.^29^ However, in 2006 only 25.3% of dentists owned an electric handpiece with or without fiber optics.^34^

High-speed handpieces are used for the majority of clinical procedures in fixed prosthodontics in North American predoctoral programs.^35^ Because electrical technology has some obvious advantages for procedures that require high-speed cutting, adoption of new technologies clearly is a critical part of student education and preparedness.^36^ Dental schools have begun to integrate electrical handpieces in their clinical settings.^32^,^36^,^37^

In 2005 the SODM decided to implement electrical handpieces for all students, while keeping the traditional air-driven handpieces technology in order to train graduates with both modalities. The students at the SODM are required to purchase standardized instruments and handpieces following an equipment list provided by the school, and they take these items into their practice after graduation.

**Figure 1.**
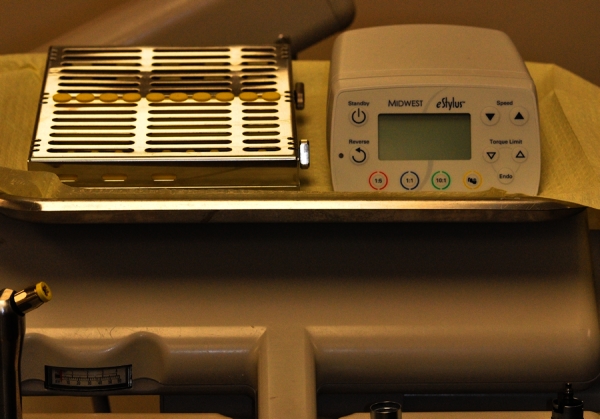
**Operatory tray with control box on the tray (right).** The silver cassette contains sterilized instruments for operative procedures.

During the first 3 years of the electrical technology implementation at the SODM, frequent but anecdotal feedback has been provided by students regarding the ergonomics of the system, frequent need for technical maintenance, and the clinical setting not being user-friendly for employment of the electrical technology. A common complaint was that control boxes required for operation of the electric handpieces were not secured and that the students were required to connect and disconnect them several times a day. Another concern was that the control box is bulky and occupies a large portion of the operatory tray (Figure 1).^28^

Because the school determined that this new technology is essential for the educational process and clearly the implementation affected the school’s primary customers, this topic was labeled as an essential process that should be addressed. When we surveyed the students and analyzed the feedback, it became evident that the current situation can be improved with the following lean tools:

1. 5S methodology that has the following subsets:
  - Sort—eliminate unnecessary items from the work-place;
  - Set in order—apply efficient storage and organizational methods;
  - Shine—thoroughly clean the work area;
  - Standardize—standardize improved practices in the work area; and
  - Sustain—commit to the new standards while constantly seeking improvement
2. Quick changeover—a structured methodology for reducing the set-up time for an activity. Set-up is defined as the preparatory task required before an activity can fulfill its intended function

Through the process, the following goals were set:

1. Create a sustainable educational and clinical environment for implementation of the technology
2. Address the complaints and concerns
3. The process should be cost neutral (preferable) or require only minimal funds from the SODM

Applying the 5S methodology, it became clear that rather than implementing electrical handpieces technology for all students, the SODM should implement it also for all operatories. The outcomes of the process were intended to eliminate items from the work area (in this case the bulky control box on the unit tray, Figure 1), create a situation in which the student can experience the same setting in the preclinical mannequin area and the main patient clinic, using the electrical handpieces without incurring an unreasonable number of complaints. It also became evident that the process of connecting and disconnecting the control boxes, which need to be carried and stored during times when students are not in the operatory, should be eliminated.

Another challenge was that several student classes had already purchased the systems, and the new setting should also accommodate the existing handpieces.

The process began with screening for companies that sell electrical handpieces (the current supplier was also invited to participate) and presenting them with the SODM’s goals. After several iterations with the suppliers, the following solution was proposed by a new vendor:

1. The vendor will equip all the operatories in the preclinical and clinical areas with control boxes integrated in the dental unit. This will allow quick connection of the handpieces without the need constantly to connect, disconnect, and store the control box while not in use.
2. The vendor will supply the SODM with adaptors that allow use of handpieces purchased by students from the previous vendor.
3. The cost for creating the new setting will be incurred by the vendor, therefore being financially neutral for the SODM.

**Figure 2.**
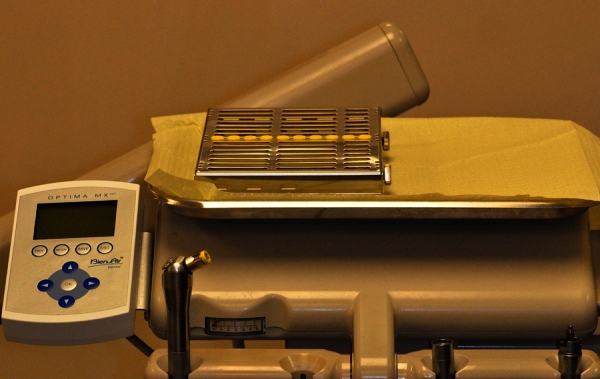
**Operatory tray with integrated control box (left).** The silver cassette contains sterilized instruments for operative procedures.

A few months after creating the new set-up (Figure 2), the students were surveyed again: the answers denoted a significantly increased level of satisfaction regarding the clinical setting—this was attributed to a significant reduction in set-up time, additional space on the unit tray, need for less storage, and simplified cleaning of the control box. Answers also showed a statistically significant decrease in the number of students who are unsatisfied with the technical service received and an increase in the number of respondents who did not have the system repaired. Interestingly, the answers showed a significant shift from those who were categorically against using electrical handpieces after graduation towards those who are “not sure.”

The results show that the intervention that was limited to replacing the control boxes influenced the overall perception of the students regarding the handpieces. The increased number of students who reported that they did not require system repairs can be attributed to the fact that the wear and tear of the new setting is significantly lower because the control box does not have to be repeatedly installed and removed, as needed in the previous clinical setting. Less service of the control box simplified the process and shortened the turnaround time for technical support and therefore decreased the number of respondents dissatisfied with the service.

This example illustrates how a process that was identified as essential can be improved with lean tools. Using these concepts, a significant impact on the primary customers was achieved, while fulfilling the goals set for the improvement process. The improvement process was done 4 years ago, and its sustainability is proved and reflected in the on-going student satisfaction with the technology and minimal maintenance requirements. Furthermore, because the process proved to be efficient and successful, other subsequent clinical projects were also addressed using similar tools.

## Abbreviations:

CODA: Commission on Dental Accreditation;
GM: General Motors;
ROI: return on investment;
SODM: School of Dental Medicine;
TMC: Toyota Motor Company;
TPS: Toyota Production System;
VMMC: Virginia Mason Medical Center.
